# Editorial: Infections and posttransplant lymphoproliferative disease after pediatric kidney transplantation

**DOI:** 10.3389/fped.2023.1221783

**Published:** 2023-06-23

**Authors:** Sarah J. Kizilbash, Blanche M. Chavers

**Affiliations:** Department of Pediatric, University of Minnesota Children's Hospital, Minneapolis, MN, United States

**Keywords:** kidney transplant, infection, PTLD, CMV, UTI – urinary tract infection

**Editorial on the Research Topic** Infections and post-transplant lymphoproliferative disease after pediatric kidney transplantation

Kidney transplantation is the treatment of choice for children with end-stage kidney disease. Compared with dialysis, kidney transplants are associated with survival benefits, improved growth, better neurocognitive development, and a higher quality of life (1–5). Despite tremendous improvements in patient and graft survival, posttransplant complications, particularly infections, continue to plague transplant recipients. Due to the increasing potency of immunosuppression, infections are one of the leading causes of morbidity and mortality in pediatric kidney transplant recipients, with a cumulative incidence of infection-related hospitalizations of 47.4% at 36 months posttransplant (6). A 2004 retrospective pediatric study, using data from the North American Pediatric Renal Transplant Cooperative Study (NAPRTCS), found a significant increase in infection-related hospitalizations from 1987 to the 2000s (7). Contrarily, infection-related mortality appears to have improved in recent years. A retrospective pediatric study of 1,810 kidney recipients, using data from the Australian and New Zealand Dialysis and Transplant Registry, found significant reductions in infection-related mortality over the last four decades (8). Transplant recipients are susceptible to numerous infections, ranging from donor-derived infections to opportunistic bacterial, viral, and fungal infections. This series discusses early detection, prevention, and management of various posttransplant infections and their complications including posttransplant lymphoproliferative disease (PTLD).

In the first paper of this series, Epperson et al. summarize the recommendations and guidelines for the prevention, diagnosis, and management of unexpected donor-derived infections in pediatric kidney transplant recipients. The authors stress the importance of detailed donors' social and medical histories in determining the potential risks of donor-derived infections. The Organ Procurement and Transplantation Network (OPTN) requires all donors to undergo testing for commonly transmissible infectious diseases, including blood and urine cultures, HIV, HBV, HCV, CMV, EBV, syphilis, and toxoplasma serology. Additional testing for endemic infections, such as strongyloidiasis, coccidioidomycosis, histoplasmosis, Chagas disease, malaria, and West Nile, may be considered for donors with a history of exposure or living in an endemic area. In recent years, COVID-19 has posed challenges for donors and recipients alike. Recommendations about COVID-19 infections continue to evolve. This paper states current COVID-19 recommendations and recommends upkeep with changing guidelines.

Urinary tract infections are frequently observed in adult and pediatric kidney transplant recipients and are the most common cause of infectious hospitalizations in children older than 5 years of age (9–11). In addition to immunosuppression, anatomic factors, such as vesicoureteral reflux and neurogenic bladder, contribute to the risk of UTIs in transplant recipients. In the second report of this series, Spiwak et al. showed that UTIs caused 30% of hospitalizations in pediatric kidney transplant recipients. Furthermore, transplant recipients were more likely to have non-*E. coli* UTI, unlike the general population, where *E. coli* constitutes 80%–90% of UTIs. This article reports data that suggest UTIs in transplant recipients are primarily due to the immunosuppression and the transplant procedure itself.

Cytomegalovirus (CMV) is a common posttransplant opportunistic infection jeopardizing patient and graft survival through invasive disease and indirect immunomodulatory effects. The manuscript by Balani et al. provides a comprehensive overview of preventive strategies (prophylaxis vs. pre-emptive therapy) and treatment modalities for CMV viremia and invasive disease. The American Society of Transplantation (AST) recommendations for the management of CMV infections are outlined in this article. These recommendations are primarily derived from adult studies, impugning their generalizability to children. The paucity of pediatric data has resulted in heterogeneous practice. A web-based survey of 13 French pediatric kidney transplantation centers, published by Madden et al. in this series, found considerable practice differences. Variations in pediatric treatment practices underscore the need for pediatric studies to determine the optimal prevention and treatment strategies for CMV in children.

PTLD, observed in 1%–3% of pediatric kidney transplant recipients, is a dreaded complication of posttransplant infections with Epstein-bar virus (EBV). Pediatric data detailing changes in the incidence of PTLD over time are lacking. Over 90% of pediatric PTLD is EBV-driven. Hence, prevention and timely treatment of EBV are the primary strategies for PTLD prevention. In this series, Cheyssac et al. studied the effect of valganciclovir prophylaxis on EBV prevention, and interestingly, found no difference in the incidence of EBV between pediatric kidney transplant recipients with and without valganciclovir prophylaxis but found an increased risk of neutropenia in patients receiving valganciclovir. Hence, immunosuppression reduction remains the cornerstone of therapy for preventing EBV and PTLD, as discussed by Fulchiero and Amaral. Studies are needed to examine the risk factors, characteristics, and outcomes of PTLD in pediatric transplant recipients.

In conclusion, posttransplant infections significantly contribute to morbidity and mortality in pediatric kidney transplant recipients. This series reviews some of the common posttransplant infections and PTLD in pediatric kidney transplant recipients and provide information on management strategies to mitigate the risk of these infections, decreasing infection-related morbidity and mortality in pediatric kidney transplant recipients.
